# *Bombyx mori Metal Carboxypeptidases12* (*BmMCP12*) Is Involved in Host Protection Against Viral Infection

**DOI:** 10.3390/ijms252413536

**Published:** 2024-12-18

**Authors:** Liang Tang, Qiong-Qiong Wei, Yu Xiao, Ming-Yan Tang, Yan Zhu, Man-Gui Jiang, Peng Chen, Zhi-Xin Pan

**Affiliations:** 1 Guangxi Key Laboratory of Sericultural Genetic Improvement and Efficient Breeding, Sericulture Technology Promotion Station of Guangxi, Nanning 530007, China; ty008tl@163.com (L.T.); 19968087382@163.com (M.-Y.T.); jiangmg888@sina.com (M.-G.J.); 2 State Key Laboratory of Resource Insects, Key Laboratory of Sericultural Biology and Genetic Breeding, Ministry of Agriculture and Rural Affairs, Southwest University, Beibei District, Chongqing 400715, China; qiongqiongwei@126.com (Q.-Q.W.); xiaoyu8230@126.com (Y.X.); zhuyan0806@126.com (Y.Z.)

**Keywords:** silkworm, BmNPV, carboxypeptidase, *BmMCP12*

## Abstract

Baculoviruses, the largest studied insect viruses, are highly pathogenic to host insects. *Bombyx mori* nucleopolyhedrovirus (BmNPV) is the main cause of nuclear polyhedrosis of silkworm, a viral disease that causes significant economic losses to the sericulture industry. The anti-BmNPV mechanism of the silkworm has not yet been characterized. Carboxypeptidase is an enzyme that is involved in virtually all life activities of animals and plants. Studies have shown that the carboxypeptidase family is related to insect immunity. There are few reports on the role of carboxypeptidase in the defense of silkworms against pathogen invasion. In this study, we identified the homologous gene *Bombyx mori metal carboxypeptidases12* (*BmMCP12*) related to mammalian *carboxypeptidase A2* (*CPA2*) and found that BmMCP12 had a Zn-pept domain. The *BmMCP12* gene was primarily located in the cytoplasm and was highly expressed in the midgut of silkworms, and the expression level in BmN-SWU1 cells was upregulated after infection with BmNPV. After overexpression of the *BmMCP12* gene, quantitative real-time (qRT)-PCR and Western blots showed that *BmMCP12* could inhibit BmNPV replication, whereas knockout of the gene had the opposite effect. In addition, we constructed transgenic silkworm strains with a knockout of *BmMCP12*, and the transgenic strains had reduced resistance to BmNPV. These findings deepen the functional study of silkworm carboxypeptidase and provide a new target for BmNPV disease prevention in silkworms.

## 1. Introduction

Carboxypeptidases (CPs) are a class of exopeptidases that specifically degrade peptide chains by releasing free amino acids one by one from the C-terminal. Carboxypeptidases can be divided into three subclasses based on their catalytic mechanisms,: serine CPs that have an active serine residue at the active site, cysteine CPs that have an active cysteine residue, and metallo-CPs that have a metal ion [1]. Carboxypeptidases play important roles in food digestion, blood coagulation, the production of growth factors, and the regulation of biological processes [2,3,4,5]. Carboxypeptidase A2 (CPA2) can be used as a characteristic marker for pancreatic cancer, specifically acinar cell carcinoma (ACC) [6]. Previous research has demonstrated that carboxypeptidase A3 is significantly overexpressed in the serum of patients infected with COVID-19, and thus, it can serve as a serological diagnostic marker [7].

The silkworm is an economically important insect belonging to the order Lepidoptera. Due to its ease of breeding, relatively large size, and well-characterized genome, it is widely used in biological sciences, biomedical applications, biomaterials, tissue engineering, and electronics [8,9,10,11,12]. However, silkworms are susceptible to various pathogens [13]. The *Bombyx mori* nucleopolyhedrovirus (BmNPV) with a circular double-stranded DNA genome belongs to the Baculovirus family [14]. The pathogen is responsible for the most common and severe form of septicemia in silkworms and thus poses a significant threat to the sericulture industry [13]. Therefore, understanding the mechanisms by which BmNPV infects its host is extremely important, and finding effective measures to prevent and control BmNPV infection is a pressing issue that needs to be addressed in the sericulture industry.

At present, 48 CPs have been identified in silkworms (34 metal CPs and 14 serine CPs), with most CPs being specifically expressed in the midgut of the silkworm, and thus, they may participate in digestion [5,15,16,17,18]. For example, MF-CPA has been reported to degrade proteins in old epidermal cells and participate in the amino acid cycle [19]. The expression of metal CPs in silkworm larvae has been demonstrated to be upregulated after infection with Bacillus bombyseptieus and BmNPV [20,21]. Therefore, we speculated that other metal CPs may participate in the response to BmNPV infection and that they may regulate the proliferation and replication of the virus.

In this study, we identified a homologous gene of *CPA2* in the silkworm database, named *BmMCP12*. To explore its role in BmNPV proliferation and replication, we analyzed *BmMCP12* through qRT-PCR, Western blotting, and immunofluorescence assays. We found that *BmMCP12* expression was upregulated in silkworm cells after infection with BmNPV. Additionally, the overexpression of *BmMCP12* effectively inhibited the proliferation and replication of BmNPV. These results strongly suggest that *BmMCP12* is involved in the response to BmNPV infection in silkworms and that it plays a role in suppressing BmNPV replication. This study provides a foundation for further analysis of the functions of metal CPs of silkworms and a basis for the analysis of the host invasion mechanisms of BmNPV.

## 2. Results

### 2.1. Analysis of the Sequence Characteristics of BmMCP12

**Figure 1:**
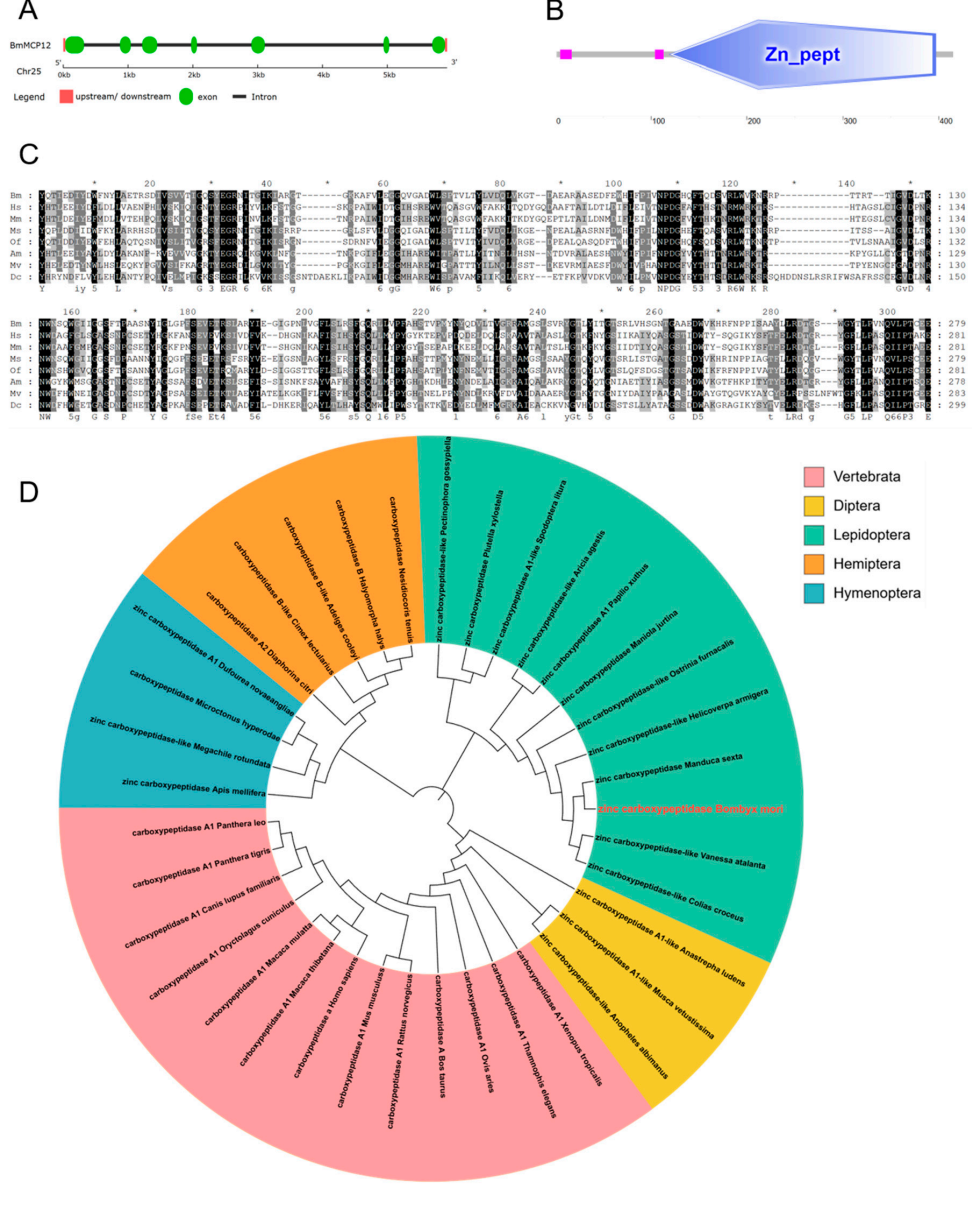
Identification of *BmMCP12* gene. (**A**) Gene structure of *BmMCP12* gene. (**B**) Prediction of BmMCP12 protein domains. (**C**) Carboxypeptidase A protein Zn_pept domain’s multiple-sequence alignment. Bm, *Bombyx mori*; Hs, *Homo sapiens*; Mm, *Mus musculus*; Ms, *Manduca sexta*; Of, *Ostrinia furnacalis*; Am, *Apis mellifera*; Mv, *Musca vetustissima*; Dc, *Diaphorina citri*. *, 10 amino acids apart. (**D**) Phylogenetic tree analysis of BmMCP12. Pink represents Vertebrata; yellow represents Diptera; green represents Lepidoptera; orange represents Hemiptera; and blue represents Hymenoptera.

To study the function of the *BmMCP12* gene, we first analyzed its sequence characteristics. The full length of the CDS of *BmMCP12* was 1248 bp, with seven exons (Figure 1A) encoding 415 amino acids determined by referring to the SilkDB3.0 database. Its domains were predicted online by SMART, which showed that it had a Zn-pept domain (119 aa-397 aa) (Figure 1B). SignalP 4.0 and TMHMM2.0 predicted the presence of a signal peptide (Figure S1A) at 1 aa-17 aa, and there was no transmembrane domain (Figure S1B). To determine whether *BmMCP12* had differences in its domain sequence from other species, we selected *Homo sapiens*, *Mus musculus*, *Manduca sexta*, *Ostrinia furnacalis*, *Apis mellifera*, *Musca vetustissima*, and *Diaphorina citri* for multi-species sequence homology comparisons. The sequence similarity to BmMCP12 varied widely among species, ranging from 29% to 69%. There was higher similarity to homologs of *Manduca sexta* but lower conservation with homologs of *Mus* (Figure 1C). MEGA6.0 software was used to construct a phylogenetic tree of BmMCP12, and the results showed that BmMCP12 was most closely related to *Manduca sexta* (Figure 1D).

### 2.2. Expression Characteristics of BmMCP12 Gene

**Figure 2:**
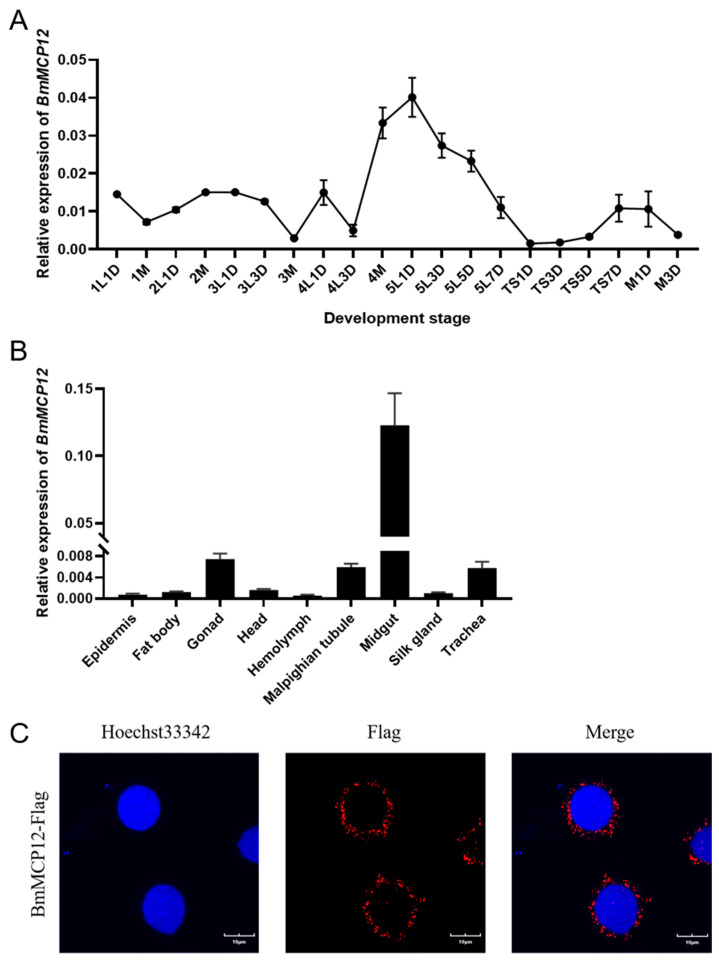
Expression patterns of *BmMCP12* gene. (**A**) Period expression analysis of *BmMCP12*. (**B**) Expression of *BmMCP12* in tissues, including epidermis, fat body, gonad, head, hemolymph, Malpighian tubule, midgut, silk glands, and trachea, of 5th-instar larvae on first day. (**C**) Subcellular localization of BmMCP12.

To study the temporal and spatial expression of *BmMCP12* in silkworms, we used qRT-PCR to detect the expression of *BmMCP12* in different stages and tissues of silkworms. The results showed that *BmMCP12* was highly expressed during the full-feeding period, reaching a peak in the fifth-instar larvae (Figure 2A) and that it was significantly highly expressed in the midgut (Figure 2B). To further clarify the subcellular localization of *BmMCP12*, a *BmMCP12* overexpression vector (Figure S2A,B) was constructed and transfected into BmN-SWU1 cells. Immunofluorescence results showed that *BmMCP12* was mainly located in the cytoplasm, with a small amount in the nucleus (Figure 2C).

### 2.3. Overexpression of BmMCP12 Inhibited BmNPV Multiplication

**Figure 3:**
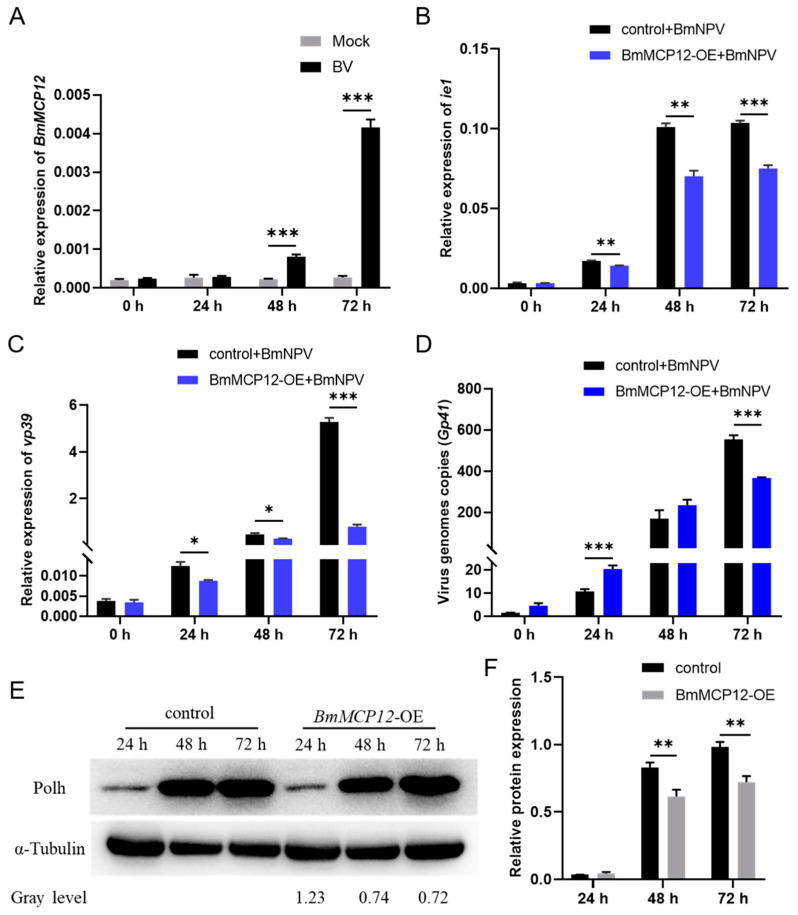
Effects of overexpression of *BmMCP12* on BmNPV replication. (**A**) Quantitative real-time (qRT)-PCR analysis of *BmMCP12* gene after BmNPV supplementation. (**B**) qRT-PCR analysis of BmNPV *ie1* gene after overexpression of *BmMCP12*. (**C**) qRT-PCR analysis of BmNPV *vp39* gene after overexpression of *BmMCP12*. (**D**) qRT-PCR analysis of genome copies of BmNPV after overexpression of *BmMCP12*. (**E**,**F**) Western blot analysis of BmNPV Polh protein after overexpression of *BmMCP12*. *** *p* < 0.001; ** *p* < 0.01; * *p* < 0.05.

Several studies have demonstrated that the CP family is related to insect immunity [22,23,24]. We thus speculated as to whether *BmMCP12* could affect the proliferation and replication of viruses. We found that *BmMCP12* expression was significantly upregulated in BmN-SWU1 cells infected with the virus (Figure 3A). To clarify the role of *BmMCP12* in viral infection, we transfected BmN-SWU1 cells with an overexpressed plasmid (BmMCP12-OE) or a control vector (control) and infected the cells with BmNPV. Quantitative results showed that the expression levels of the immediate–early gene *ie1* and the viral replication gene *vp39* were significantly decreased after *BmMCP12* overexpression compared to the control group (Figure 3B,C), and the expression of the viral gene copy number *Gp41* was also decreased compared to the control group (Figure 3D). Western blot results showed that the expression level of viral nuclear polyhedral protein PolH after *BmMCP12* overexpression was lower than that of the control group (Figure 3E,F). These results suggest that *BmMCP12* may inhibit the proliferation and replication of BmNPV in BmN-SWU1 cells.

### 2.4. Knockout of BmMCP12 Facilitated BmNPV Replication

**Figure 4:**
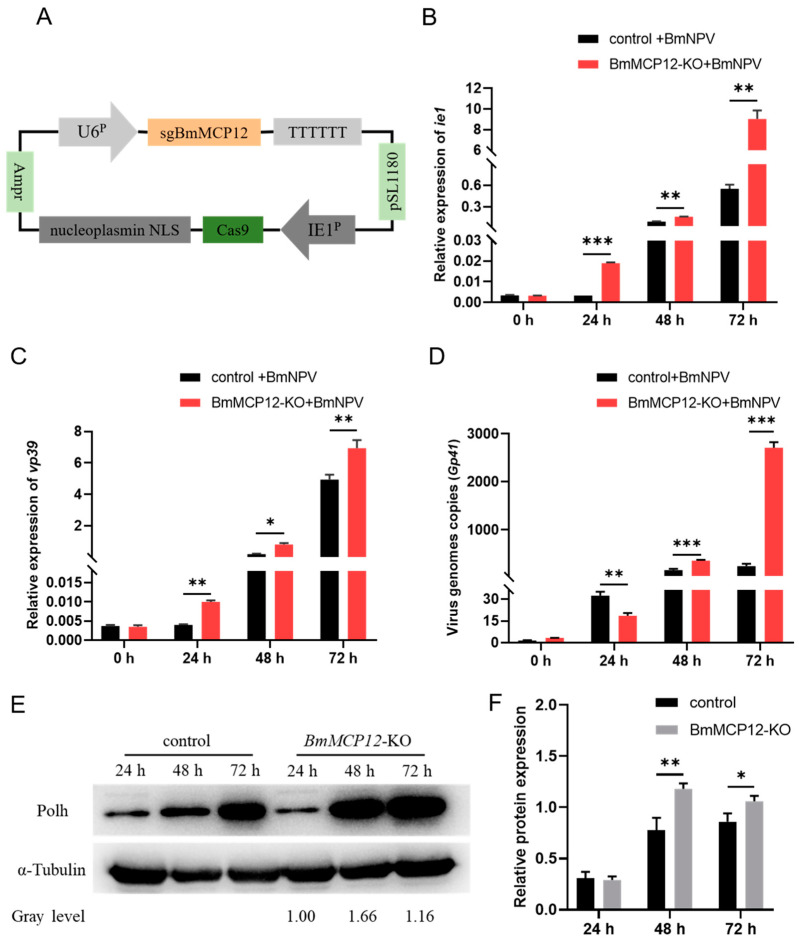
Effects of knockout of *BmMCP12* on BmNPV replication. (**A**) Schematic diagram of *BmMCP12* gene’s CRISPR/Cas9 knockout vector. (**B**) qRT-PCR analysis of BmNPV *ie1* gene after knockout of *BmMCP12*. (**C**) qRT-PCR analysis of BmNPV *vp39* gene after knockout of *BmMCP12*. (**D**) qRT-PCR analysis of genome copies of BmNPV after knockout of *BmMCP12*. (**E**,**F**) Western blot analysis of BmNPV Polh protein after knockout of *BmMCP12*. *** *p* < 0.001; ** *p* < 0.01; * *p* < 0.05.

To further identify the inhibitory effect of *BmMCP12* on viral replication in BmN-SWU1 cells, we constructed a knockout vector of *BmMCP12* (BmMCP12-KO) (Figure 4A). BmNPV was introduced after transfecting BmN-SWU1 cells with the knockout vector or a control vector. The qRT-PCR results showed that after the knockout of *BmMCP12*, the expression levels of the *ie1* and *vp39* genes were upregulated (Figure 4B,C), and the expression level of the *Gp41* gene was also higher than that of the control group (Figure 4D). Meanwhile, the Western blot results showed the opposite result compared to the overexpression of BmMCP12 (Figure 4E,F). In summary, the results demonstrated that *BmMCP12* was involved in the infection of BmNPV in BmN-SWU1 cells and that it could inhibit viral proliferation and replication.

### 2.5. Effects of BmMCP12 on BmNPV Infection at Individual Level

**Figure 5:**
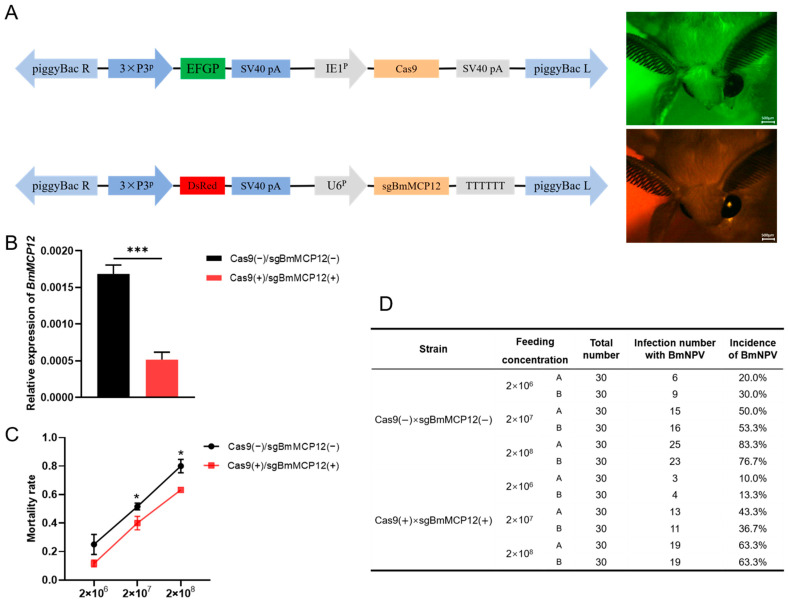
The effects of *BmMCP12* on BmNPV infection at the individual level. (**A**) The construction of transgenic lines. The plasmid containing the *enhanced green fluorescent protein* (*EGFP*) gene and the *Cas9* gene was injected into the silkworms to produce transgenic silkworm moths with green fluorescence in their eyes. The plasmid containing the red fluorescence gene (DsRed) and sgBmMCP12 was injected into silkworms to obtain transgenic silkworms with red fluorescence in their eyes. (**B**) qRT-PCR analysis showed the expression of *BmMCP12* in the transgenic knockout lines (Cas9(+)/sgBmMCP12(+)) and control lines (Cas9(−)/sgBmMCP12(−)). (**C**,**D**) Statistics on the mortality rate of the transgenic knockout lines (Cas9(+)/sgBmMCP12(+)) and control lines (Cas9(−)/sgBmMCP12(−)). *** *p* < 0.001; * *p* < 0.05.

To further investigate the function of the *BmMCP12* gene at the individual level, we created a transgenic silkworm line with a knockout of *BmMCP12* (Cas9(+)/sgBmMCP12(+)) with red and green fluorescence in the eyes of silkworm moths (Figure 5A). The qRT-PCR results showed that the expression of *BmMCP12* in the Cas9(+)/sgBmMCP12(+) strain was significantly reduced compared to the Cas9(−)/sgBmMCP12(−) strain (Figure 5B). The fourth-instar larvae of the Cas9(+)/sgBmMCP12(+) and Cas9(−)/sgBmMCP12(−) transgenic strains were challenged with viruses at concentrations of 1 × 10^5^, 1 × 10^6^, and 1 ×10^7^ OBs/head, and the numbers of dead larvae were recorded. The results showed that at the concentrations of 1 × 10^6^ and 1 × 10^7^ OBs/head, the mortality rate of the Cas9(+)/sgBmMCP12(+) transgenic strain was higher than that of the Cas9(−)/sgBmMCP12(−) group (Figure 5C,D). The results indicated that the KO strain of *BmMCP12* facilitated BmNPV infection.

## 3. Discussion

Baculoviruses are intracellular parasites that are specific to invertebrates. Their replication depends on the life activities of the host [25]. Baculoviruses can be divided into granular viruses (GVs) and nuclear polyhedrosis viruses (NPVs). BmNPV is a typical baculovirus that infects silkworms and causes significant economic losses to the sericulture industry. There is no effective drug for the prevention and control of BmNPV disease. Therefore, understanding the mechanism of BmNPV infection and the creation of BmNPV-resistant silkworm strains can prevent and control BmNPV infection [26]. Research on silkworm resistance to BmNPV has identified genes and proteins with antiviral effects. The Bombyx mori Ser/Thr protein phosphatase 2A(BmPP2A), BmSTING, and G protein β subunit 1 (BmGNβ1) can effectively inhibit the replication of BmNPV after infection in silkworm cells [27,28,29].

Proteases can be divided into endopeptidase and exopeptidase according to their mode of action, and exopeptidases can be divided into CPs that degrade and release amino acids one by one from the C end of the peptide chain and aminopeptidases that begin from the N end of the peptide chain [30]. Metal CPs are the most widely studied and the most abundant CPs. In mammals, CPs can be used as a marker for the early diagnosis or a prognostic risk indicator of cancer [31,32,33,34]; CPs have potential links to both inflammatory and protective markers [35]. In insects, researchers have found that *carboxypeptidase D* (*CPD*) is associated with the development of *Drosophila* wings [36]. *Carboxypeptidase A* in *Anopheles stephensi* can effectively reduce the infection rate of the malaria protozoa and block its development in the midgut, and thus, it can be used as a candidate drug for the blocking transmission vaccine (TVB) [37]. These results suggest that CPs may be involved in host resistance to viruses.

In *Bombyx mori*, 48 CPs have been identified, far more than in other species [17]. The identified genes of silkworm carboxypeptidase can be classified into four categories, including the S12 subfamily containing the S12 domain and the S28 subfamily containing the S28 domain, belonging to the serine CPs, and the M12 subfamily containing the M12 domain and the M14 subfamily containing the M14 domain, belonging to the metal CPs [15]. Carboxypeptidases are involved in the processes of digestion and molting in silkworms. The expression levels of *BmSCP1* and *BmMCP30* in silkworms were significantly higher after repeated feeding than in a normal feeding group [15]. *BmCPA* is gradually enriched during the molting of silkworms, and it regulates the molting process together with *BmCPAi* [38]. Research has shown that CPs are highly expressed in the midgut [15]. The midgut is the first barrier to BmNPV infection in silkworms, and it is also important in resistance to infection [39]. However, there are few reports concerning the role of CPs in the defense against pathogen invasion. *BmMCP12* is homologous to the mammalian *CPA2* gene. The results of tissue and period expression pattern analyses showed that *BmMCP12* was highly expressed in the midgut and in larvae at the full feeding stage, especially in the fifth-instar larvae (Figure 2). The larval peak feeding period is the main period of energy acquisition. In Lymantria dispar, 48 h fourth-instar larvae showed increased resistance to baculovirus compared to 0 h fourth-instar larvae [40]. These results suggested that *BmMCP12* may have antiviral activity. After BmNPV infection, the expression levels of *BmMCP12* showed an increasing trend with time, indicating that the host could upregulate the expression of *BmMCP12* to prevent the replication of BmNPV.

Upregulating *BmMCP12* by overexpression showed that the proliferation and replication of BmNPV were significantly decreased (Figure 3); conversely, the knockout of *BmMCP12* reversed these effects (Figure 4). Hence, we created the knockout transgenic strain Cas9(+)/sgBmMCP12, and found that the transgenic strain had lower resistance to BmNPV than the control strain (Figure 5). These results indicated that *BmMCP12* could effectively inhibit the replication of BmNPV in *Bombyx mori*. Concerning the mechanism of *BmMCP12* protecting the host against viral infection, previous studies have shown that CPs can directly target to virus-related proteins and reduce viral infection and inhibit viral replication [37,41]. Moreover, CPs may play an antiviral role by regulating the host’s immune response [22]. In the case of novel coronavirus infection mediated by CD16 and ACE2 receptors, human macrophages activate inflammasomes, release IL-1 and IL-18, and cause the pyroptosis of infected macrophages, thereby interrupting the viral cycle [42]. Therefore, we speculate that *BmMCP12* may inhibit the replication of the virus by regulating the immune response of silkworms, but this hypothesis needs to be verified by further experiments.

In conclusion, we have demonstrated that *BmMCP12* is highly expressed in the midgut of silkworms and that it is involved in the response of silkworms to BmNPV, inhibiting the proliferation and replication of the virus. Therefore, we can create transgenic strains with high resistance to BmNPV through the overexpression of *BmMCP12*, thereby aiding the sericulture.

## 4. Materials and Methods

### 4.1. Silkworm Strains, Cell Lines, and Viruses

The silkworm strain DaZao and the cell line BmN-SWU1 were provided by our laboratory. The cell line BmN-SWU1 derived from the *B. mori* ovary was cultured at 27 °C in TC-100 medium (United States Biological, Swampscott, MA, USA) supplemented with 10% fetal bovine serum (BIOAGRIO, Mountain View, CA, USA), 100 U/mL of penicillin, and 100 μg/mL of streptomycin (Gibco, Grand Island, NY, USA). Occlusion bodies (OBs) were obtained from the silkworm larvae infected with the virus and purified through gradient centrifugation. The original solution of the virus was stored at 4 °C.

### 4.2. Quantitative Real-Time (qRT)-PCR

Total RNA was extracted from tissues and cells using a Total RNA Kit (Omega Bio-Tek, Norcross, GA, USA). The RNA was reverse-transcribed to cDNA using a Prime Script™ RT Reagent Kit (TaKaRa, Dalian, China). qRT-PCR was performed using the Hieff^®^ qPCR SYBR Green Master Mix (Yeasen, Shanghai, China) and the CFX96™ Touch Real-Time PCR System (Bio-Rad, Berkeley, CA, USA). The reaction conditions were 95 °C for 30 s, followed by 40 cycles of 95 °C for 5 s and 60 °C for 30 s. Three repetitions were performed for each sample. The eukaryotic translation initiation Factor 4A (SW22934) was used as the internal reference gene. The primers are listed in Table S1.

### 4.3. Plasmid Construction

To construct an overexpression plasmid for the *BmMCP12* gene, amplification primers with restriction sites were designed, and specific sequences were amplified using the cDNA library and then inserted into the eukaryotic insect expression vector pIZ/V5-His. The SgRNA of the *BmMCP12* gene was predicted using the CRISPR direct website (http://crispr.dbcls.jp/, accessed on 12 October 2021) and synthesized by BGI (Beijing, China). The synthesized fragment was attached to the pSL1180-ie1-Cas9-U6 vector to obtain the recombinant plasmid. The primers are listed in the Supporting Information, Table S1.

### 4.4. Transient Transfection and Viral Infection

The constructed plasmid was transfected into BmN-SWU1 cells by TransIT^®^-Insect Transfection Reagent (Mirus, Madison, WI, USA) according to the manufacturer’s instructions. BmNPV was added to the cells after 48 h of transfection. The multiplicity of infection (MOI) of BmNPV was defined as 1, and the time of initial infection was recorded as 0 h. Samples were collected at specified time points for subsequent experiments.

### 4.5. Immunofluorescence Assay

The BmN-SWU1 cells were seeded in a 24-well plate (Corning, NY, USA) with coverslips (Fisher Scientific, Waltham, MA, USA). After 24 h, the cells were transfected with the plasmid, and the cell culture medium was removed after 48 h of transfection. The cells were fixed with 4% paraformaldehyde for 30 min and then with 0.1% Triton X-100 permeabilization for 10 min. After each treatment, the cells were washed with phosphate-buffered saline (PBS) and then blocked with PBS containing 10% goat serum and 3% BSA for 1 h. The cells were incubated with a mouse flag-tagged antibody for 2 h at 37 °C and then washed six times with PBS. Alexa Fluor 555-conjugated goat anti-mouse IgG and Hoechst 33342 (Life Technologies, Carlsbad, CA, USA) were added and incubated for 1 h at 37 °C and then washed with PBS six times, for 5 min every time. The treated cells were imaged using a laser scanning confocal microscope (Olympus, Tokyo, Japan).

### 4.6. Western Blot Analysis

BmN-SWU1 cells were inoculated in six-well plates and infected with the virus after being transfected for 48 h. The cells were collected after 0 h, 24 h, 48 h, and 72 h, and Western lysis buffer (Beyotime, Shanghai, China) was added to the cells. After 2 h, the cells were centrifuged at 13,000× *g* for 10 min; the protein supernatant was collected with 5× SDS loading buffer (Beyotime, Shanghai, China) and boiled for 10 min. After SDS-PAGE, the denatured proteins were transferred to a polyvinylidene fluoride (PVDF) membrane (Millipore, Burlington, MA, USA), blocked with 5% skimmed milk powder at room temperature for 1 h, and then incubated with a primary antibody for 2 h at room temperature. The membrane was washed six times with TBST and then incubated with a secondary antibody for 1 h; after that, it was washed with TBST six times. Finally, the results of the Western blots were analyzed with the ECL Western blotting Detection System (Bio-Rad, Hercules, CA, USA).

### 4.7. Construction of Transgenic Strains

The SgRNA of *BmMCP12* was connected to the piggyBac [3 × P3DsRed] vector. The constructed plasmid was microinjected into non-diapause silkworm eggs, screened in silkworms with red fluorescence in their eyes, and then hybridized with transgenic silkworms expressing the Cas9 protein. Finally, the transgenic silkworms with *BmMCP12* knockout were obtained by screening for the simultaneous expression of red and green fluorescence together; the Cas9(+)/sgBmMCP12(+) transgenic strain was a successful construction.

### 4.8. Mortality Rate Analysis

The Cas9(+)/sgBmMCP12(+) and Cas9(−)/sgBmMCP12(−) transgenic strains were fed to the fourth instar. The fourth-instar larvae were then divided into three groups of 30 larvae each and then infected with viruses at concentrations of 1 × 10^5^, 1 × 10^6^, and 1 × 10^7^ OBs/head. The number of dead larvae were recorded every day.

### 4.9. Statistical Analysis

Statistical analysis was performed using GraphPad Prism 8 (Graph Pad, San Diego, CA, USA). The *t*-test was used to evaluate the differences between treatment groups. *p* < 0.05 indicated a significant difference and *p* < 0.01 indicated an extremely significant difference, which were denoted by “*” or “**”, respectively. Data are presented as the mean ± SD of at least three independent biological replicates.

## Data Availability

The original contributions presented in the study are included in the article/Supplementary Material, further inquiries can be directed to the corresponding author/s.

## Supplementary Materials

The following supporting information can be downloaded at https://www.mdpi.com/article/10.3390/ijms252413536/s1.
