# Supplementary material for: Bombyx mori Metal Carboxypeptidases12 (BmMCP12) Is Involved in Host Protection Against Viral Infection
Source: Int J Mol Sci. 2024 Dec 18;25(24):13536. doi: 10.3390/ijms252413536 (PMC11677143; doi:10.3390/ijms252413536)
Supplement: Supplementary file 1 [file ijms-25-13536-s001.zip › ijms-3281577-supplementary.pdf]

Supplementary Materials:

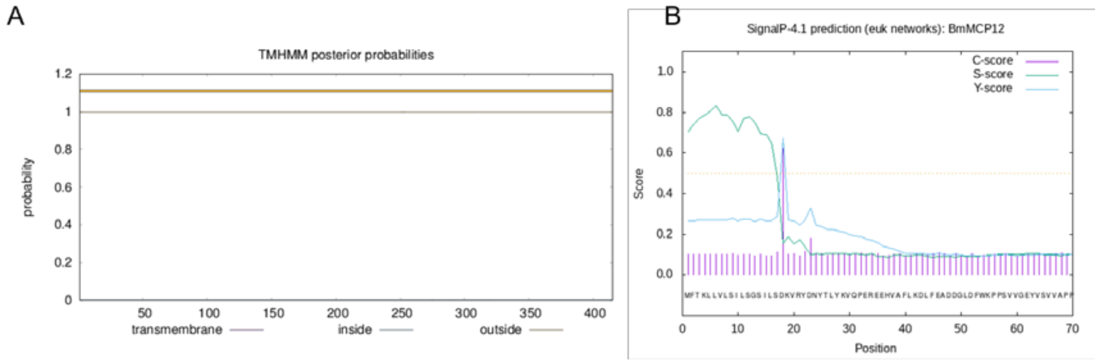

Figure S1. Protein primary structure analysis of the BmMCP12. (A)Transmembrane domain prediction of the BmMCP12. (B) Prediction of signal peptides of BmMCP12.

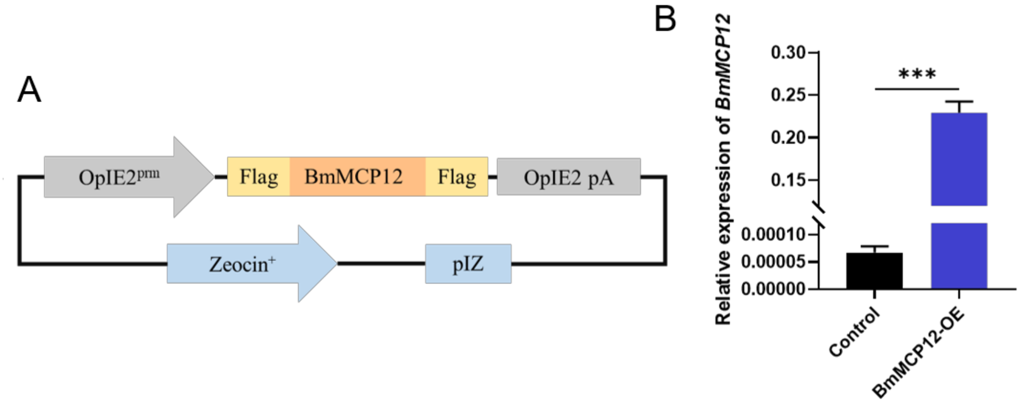

Figure S2. Construction of overexpression vector of the *BmMCP12*. (A) Verification of the effect of the overexpression vector; (B) Schematic diagram of the *BmMCP12* gene overexpression vector. *** $p<0.001$

Tabel S1. Sequences and names of primers used in this study

name	Sequences
BmMCP12-F	CACTTTACATCACTGGCACTTC
BmMCP12-R	CGGAGTAAGTATGCTGCAGATA
SW22934-F	TTCGTACTGGCTCTTCTCGT
SW22934-R	CAAAGTTGATAGCAATTCCCT
BmMCP12-EcoR I -Flag -F	CGGAATTCATGGATTACAAGGATGACGACGATAAGTTCACGAAACTATTGGTAC
BmMCP12- Xho I -Flag -R	CCGCTCGAGTTACTTATCGTCGTCATCCTTGTAATCCAGTATGTTAATAAATTTAGCT
sgBmMCP12-F	ATTAAAATTGCTCGGGGCAC
sgBmMCP12-R	GTGCCCCGAGCAATTTTAAT
